# Medical and Philosophical Intelligence

**Published:** 1819-02

**Authors:** 


### MEDICAL AND PHILOSOPHICAL INTELLIGENCE.

*Essays on the proximate Mechanical Causes of the general Phenomena of the Universe;* by Sir RICHARD PHILLIPS. 12mo. pp. 96. London, 1818.

ON introducing to our readers a new system of physical philosophy, we are disposed, in the first instance, to make some observations in reply to the objections that may, perhaps, be made by some persons to the insertion of such a subject in a work professedly devoted to medical science; and also to our adducing, as a matter worthy of serious consideration, a theory of the nature of the universe, which is expressly in opposition to that which has for so long a period been generally received with passive approbation, is supposed to be founded on evidential truth, and to explain in a satisfactory manner all the pheuomena to which it relates.

With respect to the former of these objections, we will only observe, that we adopt the sentiment of Hippocrates,—that a knowledge of natural philosophy is an essential qualification among those which tend to form a good physician. An attentive consideration of the annals of science will vindicate the propriety of our conduct with respect to the latter, by showing that it is not the domination of opinions for long periods of time, that should deter men from opposing to them such as appear to be more rational, and raised on a more evident and intelligible basis. It is not for us to determine whether or not this may be the case with respect to those we are about to consider: they, however, evidently merit the attention of philosophers; and, therefore, we judge that to lay them before our readers is part of our duty in "the fulfilment of our bond."

It may be salutary advice to those persons who are disposed to treat with contempt a theory of the nature of the universe which is adverse to that of NEWTON, to request them to consider that the wisest men in all ages have been influenced by the prejudices and superstitious errors of their time: and which, from their having been impressed on the mind from the earliest dawn of reason, and supported by the general authority of their contemporaries, have been admitted without examination, and made to form the bases on which arguments have been raised as on the most evident and solid foundation. The age coeval with the life of Newton was one in which the most visionary notions respecting physics reigned in Europe without control: it was an age in which natural magic, sympathies, and other ridiculous occult influences, were admitted by the most eminent philosophers; and to many of whom, indeed, the observation of BACON, "*ubi diligentia defuit, spes superfuit,*" was too strictly applicable; and, as he continues to remark, "*præcepta enim magiæ naturalis talia sunt, ac si considerent homines terram subigere, et panem comedere absque sudore vultus*; *et par otiosas et faciles corporum applicationes rerum potentes fieri*: or else they, with as little piety as true philosophy, on every occasion, "*tum inquirendi, tum operandi, proposuit Deus.*" In such a school was Newton educated.

The causes of the approach of bodies to each other in space, and the fall of bodies and the return of projectiles to the earth, (which phenomena have been designated by the terms *attraction* and *gravitation*,) were considered by some of the Greek philosophers as dependent on certain innate principles of matter, which acted by means of attractive effluvia or emanating particles given off from bodies; and these notions, Sir RICHARD PHILLIPS

observes, had been again promulgated in Europe for a considerable period previous to the time of Sir Isaac Newton. It was this philosopher, however, who applied geometry to investigate the detail of those phenomena according to the law of emanations in general; but he left our knowledge of the proximate cause where he found it; he spoke of attraction and gravitation as inherent principles of all matter, and used the same terms to express the tendency of bodies to fall together.

"We might forbear (says Sir Richard Phillips) to consider gravitation except as a totality producing a given result, were it not that the Newtonian philosophy, in applying it to the solution of the planetary motions, considers it as a *specific* centripetal force, whose effect it would be to consolidate the universe in a mass, but for *another specific* force, which has been denominated the *projectile* force. It is evident, therefore, that, in inventing this latter force, and introducing it into his system of physics, Sir Isaac Newton considered *gravitation* itself as a substantive force, acting in one direction, and not simply as a name of the totality of the phenomena, as many of his commentators would infer."

These forces Sir Isaac Newton considered as acting simultaneously at right angles with respect to each other: by these laws he found he could explain the curvilinear motions of the planets; and then, from those curvilinear motions, he inferred the existence of such laws!

"To minds accustomed to contemplation and original thinking, (continues Sir Richard Phillips,) it can scarcely be necessary to expose the absurdities of the doctrine of *innate* attraction. If any one examine the principle that a body acts on another in a place where it is not,—without any intervention or contact being supposed to be necessary,—he will feel ashamed of the philosophy which adopts it. Till Mr. Locke examined the doctrine of *innate* ideas, philosophers were, however, not ashamed of conferring *innate* properties as often as phenomena required them."—"In regard to the mechanical cause of gravitation, there have been many hypotheses; but all have failed, either in probability or in agreement with the law of the force. The relative force being always governed by the law of emanation in odours, light, and heat, the principle of gravitation has often been considered as analogous to that of emanation; and, by a false analogy, some species of emanation have, therefore, been considered as the sufficient cause. What, however, is an emanation but a flow of particles from a centre towards a concave surface,—a principle of action calculated to repel, or drive away, rather than attract, or draw towards?"—"Yet, however great may be the difficulties of the various attempts to solve the phenomena of gravitation, none have ever been made to account for the *projectile force*. Taking its existence for granted, all Newton's commentators agree that it must be the proximate power of the Deity!"

Instead, however, of a system which has introduced into science such fanciful and arbitrary forces, the author of these Essays is desirous of establishing a system of physics founded on the universal and analogous principle of Motion, as it is transferred, by various combinations, from the greatest to the smallest portions of matter. He is desirous of proving that all phenomena of matter are effects of *motion*; and of making it appear that existing motions are competent to produce all known phenomena.

"*Motion* (he continues to observe) is that universal principle which confers on masses of matter the power of acting on other masses. In regard to matter, which is essentially inert, it is the source of momentum or potentiality, and is the animating soul of the universe. *Space* is the stage, *matter* is the subject, and *motion* is the agent producing all phenomena. As it affects atoms, it produces various densities; as it affects aggregates, it creates varied organizations; and, as it affects different aggregates, it developes the relative properties of matter.

"*Motion*, as it is transferred from body to body, from great to small, or from small to great, necessarily creates and constitutes a system of universal action, producing on inert matter a simultaneous system of universal and co-equal *re-action*. From this effect of action and re-action flows, in like manner, a necessary *balance of all natural forces*, however complicated may be the machinery on either side of the fulcrum of any system: and a further result of that balance is an universal fitness, correspondence, harmony, and analogy, of mechanical causes and effects."—" Matter has power or momentum when motion is communicated to it; but, as any motion, which has been communicated to any body, is soon transferred to the surrounding matter, so, when the motion has departed, the body ceases to have power or momentum."

The explanation given by Sir Richard Phillips of the phenomena ascribed to the power termed gravitation in the Newtonian system, does not interfere with any of the known relations of bodies, as determined by experiments and observation, or as applied in the geometrical and analytical investigations of Galileo, Kepler, Des Cartes, Newton, Euler, La Grange, Herschel, or La Place; because the law of the forces is the same on this theory as on that of Newton—that the force of transferred motion is directly as the quantity of matter, and inversely as the square of the distance.

In illustration of this theory, Sir Richard Phillips enters at some length into the cause of the return of a projectile, when of a certain density, to the earth; which, he would demonstrate, depends on the orbicular and rotatory motions of the earth, by which all bodies on it are influenced. The rotatory motion of the earth produces an uniform orbicular velocity of bodies of various density in *circles of different radii*: by this means, media of *different densities*, which, being subject to a common force, and consequently receiving an equal momentum, would otherwise have different degrees of orbicular velocity, are made to keep an equal and uniform pace in the orbit of the earth. It is this rotatory motion that reduces to order what otherwise would be chaotic. Hence it is that all fluids are impelled into a level surface; hence too, doubtless, it has been that masses of the same density have formed themselves into strata while in a state of solution; hence arise all the phenomena which result from any disturbance in the order of density; and hence it is that, when a heavy body is thrown into lighter fluids, or a light body into dense fluids, the general law is proved by phenomena of ascent into circles of enlarged rotation, or descent into circles of smaller rotation, proportioned to the density.—" Every one who understands the terms (the author remarks,) is likely to feel as an axiom that the orbicular and rotatory motions of the earth necessarily give weight to bodies and laws to their fall, because the moving masses are in contact, and are so many patients partaking of the general motions."

Thus, a body, suddenly elevated from an inferior circle of rotation into one where a more rapid motion exists, or where a motion exists which does not accord with the density of the elevated body, is necessarily repelled from superior strata to inferior strata, till it finds its due level, or balance of motion and density, or till it finds support above its due station, on the concrete or fixed masses of the earth's surface. And thus, if a ball were projected at any height in the earth's atmosphere, with the velocity of that portion of the atmosphere, it never would have any tendency to fall to the earth, according to the system of Sir Richard Phillips, as well as that of Newton.

Galileo, who was the first geometrician who analysed the phenomena of falling bodies, considered that bodies thrown perpendicularly upward, merely describe in rising and falling the same straight line. But, it appears evident, were this the case, that bodies projected perpendicularly from it would not return to the same spot from which they parted, since the earth in one second of time would have been carried forward a distance of 100,000 feet.—Sir Richard Phillips would infer, that an upright projectile does not describe a perpendicular line, but two sides of an obtuse triangle: the direction to which course is given by the rotatory motion of the earth, and this course is afterwards influenced in proportion to its density, by the different media, through which it must pass: until, having lost its projectile force, and the medium to which it had been carried, being incapable of urging it with increased velocity, it would necessarily fall, if more dense, or be driven into the circle of its own velocity, with a force equal to the slight nascent deflection required to return it to the spot from which it was projected. The influence of the rotatory motion of the earth on projectiles, is illustrated by the dropping of a ball from the top-mast of a ship, which at the time was moving at any given rate, when the ball will fall perpendicularly at the foot of the mast, exactly as though the ship had been standing still. The fall of a ball at the foot of a tower, when dropped from its top, is also adduced in support of the principles of this system.

The above is an abstract of the doctrine of Sir Richard Phillips respecting the phenomena of the earth; and many powerful arguments are adduced by this philosopher to show, that those of the universe may be explained on the same principles; our limits, however, will not permit us to enter into the consideration of them; we can only adduce the following observations of the author on this part of his theory.

"The phenomena of the universe thus appear to be results of a system of motion transferring motion, or of motion generated by motion. By this simple agency a stone is propelled to a planet by the motions of the planet—a planet is carried round the sun by the motions of the sun—a secondary is carried round a primary by the joint motions of the sun and primary—and the motions of the sun are, perhaps, caused by the motions of systems of suns—while the motions of those systems may again be caused by other superior motions! In short, all nature consists of a series of included motions, produced by the motions of superior bodies and systems, till we ascend to the first term in the series—to some inscrutable CAUSE of CAUSES!"

Those who may not be disposed to admit evident truth in this theory, must at least acknowledge its sublimity—it simplifies nature—It explains all phenomena according to the known laws of the agency of matter, without the intervention of any imaginary occult principle—The general mathematical laws of it are the same as those heretofore determined; although arrived at by different trains of argument; and apparently founded on evident causes; and therefore, if true, must improve all our reasonings in regard to them, and lead to valuable discoveries by their applications to various subjects both in theory and practice.

The judicious advice adduced by Lucretius, on terminating his exposition of the doctrine of Epicurus, may not be unworthy of consideration on the present occasion; and with that we shall terminate this subject.

"Desine quapropter novitate exterritus ipsa

Exspuere ex animo rationem: sed magis acri

Judicio perpende: et si tibi vera videtur,

Dede manus: aut, si falsa est, accingere contra."

Some accounts have been published by Dr. Allbin, of Constantinople, and Dr. Laford, of Salonichi, to show that vaccination has the power to prevent the susceptibility to the infection of the plague. It is stated that, of six thousand persons vaccinated at Constantinople, not one became affected with the disease during a period when it was prevalent; and also that the Armenians are described as being entirely free from it, in consequence of having recourse to this measure. We have thought it proper to mention these circumstances; but we must remark, that the accounts are not related with sufficient precision to lead us to attribute much importance to them. No decided inferences can be drawn from the fact of such a number of persons having escaped the malady during its prevalence, after they had undergone vaccination; unless it could be ascertained whether or not their bodies had come in actual contact with those of persons affected with that disease, or with substances that might have been embued with the perspirative matter, &c. of their skin; for it is now nearly satisfactorily ascertained that that is the only way by which the infection can be communicated.

---

The application of a ligature to the common carotid artery, for the cure of aneurism of that vessel, has lately been performed with success in St. Bartholomew's Hospital. The patient was a middle-aged man, and had apparently suffered from the disease about two years. No unpleasant symptoms followed the operation.

---

The following letter has been addressed to many gentlemen of the medical profession, and we think the promulgation of it may not prove unacceptable to the faculty in general.

"*Lunatic Asylum, Hoxton House, Hoxton;*
*January* 1, 1819.

"Sir,

"Permit me to inform you that my house, which has been established more than a century, continues to receive patients afflicted with mental derangement.

"The successive improvements which have been made in my establishment, at a very considerable expence, has rendered it peculiarly adapted for the treatment of this malady, by uniting comfort and security in a healthful situation. It is my wish to impress and satisfy every medical practitioner, that any patient he may think proper to confide to my care will be considered *as under his exclusive treatment,* and that the management he may direct will be, in every instance, followed and strictly complied with.

"For the attention and humane care which has ever distinguished my establishment, I beg leave to appeal to the commissioners of the Royal College of Physicians; whose impartial report has ever been the highest sanction to my unremitting endeavours to exemplify, by tenderness of treatment, cleanliness, excellent diet, and general diffusion of comfort, the comparative superiority of this asylum.

"I have on my premises accommodation for all classes of patients, and (if required) separate houses and airing-grounds for those of a superior situation in life; and can, at an hour's notice, send careful and attentive male and female servants to take charge of persons labouring under mental derangement, either in London or any part of the country.

"I am, Sir, very respectfully,

"Your most obedient servant,

"Jonathan Miles."

---

*Prize Questions.*—The Society of Sciences of Haerlem has proposed the following questions for consideration, previous to January the 1st, 1820.

1. "How far has it been demonstrated that the fumigations by chlorine, as directed by Guyton-de-Morveau, have prevented the spreading of contagious maladies? What are the contagious diseases in which the effect of this gas deserves to be tried; and what ought to be observed in such experiments? Is there any reason to expect a more salutary effect in the prevention of contagion, from any other means hitherto employed or proposed?

2. "What are we to regard as distinctly proved in respect to the gastric juice of the human body, and its influence in the digestion of food? Is its existence sufficiently proved by the experiments of Spallanzani and Senobia; or has it been rendered doubtful by the experiments of Montegre? What is it that comparative anatomy, and principally the opening of the stomach of animals killed either after fasting, or in a short time after having taken food, has rendered probable in this respect? And, in the case of the existence of the gastric juice in the human body being regarded as a fact perfectly established, what ought we to avoid in order not to impair its effect in the process of digestion?"

The premium for the best dissertation on each of those series of questions is a gold medal, or 150 florins.

---

The Royal Academy of Sciences of Paris has announced that a gold medal of 440 francs value will be given to the author of that printed work, or manuscript, which shall appear to have contributed most to the progress of experimental physiology. The dissertations are to be transmitted before the 1st of December, 1819.

### REPORT OF DISEASES.

THE subject which should engage our chief attention in a report of the prevalent diseases of the metropolis, is that of fever; a form of disease of the highest importance at all times, but particularly so at the present period, when so much anxiety has been evinced respecting the extent of its existence and the mode in which it is disseminated. A somewhat more than ordinary proportion of cases continues to occur, especially in the practice of the physicians to public institutions; but that form usually designated typhus is almost exclusively confined to the lower classes of people: a circumstance from which arguments have been deduced in opposition to its contagious nature, by those who consider it to arise from common causes, the effects of which are modified by particular habits of life. It is not, of course, our intention to discuss this point here; but we must observe, that facts are occasionally witnessed which should determine its contagious property, according to the most correct rules of argument. The character which this form of disease has assumed in a great proportion of instances has been somewhat formidable, from the combination of severe inflammation of the respiratory organs with extreme debility. This debility has, in some instances, appeared to depend on cerebral congestion; in others, on the consequences of previous excitement; but, in a great proportion, on causes apparently inexplicable, or which are not immediately connected with the disease. Affections of the second class most frequently arise from the want of efficient medical assistance in the early stages of the malady. In this state, especially, *signs* have a pre-eminence over *symptoms* in assisting us to form the diagnosis; for the latter, which Galen happily compared to the shadow of a body, are here so obscured as to be with great difficulty distinguished with precision. These cases particularly demand a judicious and active mode of treatment. To urge the propriety of the use of the lancet in a great proportion of them is, we hope, unnecessary at the present day.

Scarlatina has, within the last few days, began to appear in an epidemic character.

Apoplexy and paralysis continue to prevail to an unusual extent, even for the season of the year. Warm winters will, we believe, be found to have been particularly productive of this malady. It has, in many instances, been preceded with dyspeptic affections, with which it appeared to be connected rather as a cause than as an effect. A peculiar cleanness and tremulous motion of the tongue was particularly observed, in one case, for some days previously to a fatal attack of this kind.

Rheumatism, cynanche, catarrh, and inflammation of the respiratory organs, are, as usual at this season, an urgent class of diseases. Consumptive patients, who, from the mildness of the season, are induced to appear much in the open air, have lately made more than ordinarily rapid progress to that state where even hope deserts them. Is there no physician of the present day, whom observation and experience will induce to rise up to vindicate the efficacy of digitalis in the alleviation of a great proportion of the cases of this malady? a remedy once so celebrated, but now, we fear, almost entirely neglected by practitioners in general.

The less immediately dangerous, though important, diseases which appear to predominate, are dyspepsia and gravel. The former may be attributed to the resort to the metropolis of those persons who are expressly the subjects of it. We have witnessed the adoption of Dr. Magendie's mode of treatment for the latter, in a few instances, with some advantage; but the patients could not be persuaded to a restriction of regimen to the extent directed by that physician, and therefore they cannot be fairly adduced as specimens of the full extent of what might be its efficacy in the cases to which it was here applied.

### METEOROLOGICAL JOURNAL,

**By Messrs. William Harris and Co. 50, Holborn, London.**

***From the 20th December to the 19th January, 1819, inclusive.***

| Day of Month. | Moon. | Rain gauge | Therm. | | | Barom. | | De Luc's Hygrom. Dry | Damp | | Wind. | | Atmospheric Variation. | | |
|---|---|---|---|---|---|---|---|---|---|---|---|---|---|---|---|
| Dec | | | | | | | | | | | | | | | |
| 20 | ● | | 45 | 49 | 47 | 30·12 | 30·01 | | 10 | 12 | SSW | WSW | Clo. | | Fine |
| 21 | | | 48 | 47 | 30 | 30·07 | 30·38 | | 10 | 11 | W | WNW | Clo. | | Fine |
| 22 | | | 32 | 35 | 30 | 30·48 | 30·48 | | 11 | 10 | NW | WNW | Fog | | |
| 23 | | | 30 | 35 | 28 | 30·39 | 30·27 | | 11 | 11 | ENE | E | Fog | | |
| 24 | | | 30 | 34 | 27 | 30·27 | 30·21 | | 12 | 10 | ESE | SW | Fog | | |
| 25 | | | 30 | 33 | 35 | 30·11 | 29·97 | | 13 | 10 | SSE | SSE | Fog | Clo. | |
| 26 | | | 36 | 37 | 29 | 29·99 | 29·95 | | 15 | 11 | S | SE | Clo. | | |
| 27 | ◐ | | 30 | 36 | 35 | 30·21 | 30·32 | | 10 | 10 | NE | NE | Clo. | | |
| 28 | | | 38 | 44 | 32 | 30·47 | 30·53 | | 9 | 10 | NE | N | Clo. | | |
| 29 | | | 35 | 38 | 30 | 30·60 | 30·60 | | 9 | 8 | W | W | Fine | Clo. | |
| 30 | | | 32 | 35 | 30 | 30·51 | 30·43 | | 8 | 9 | W | W | Fog | Clo. | |
| 31 | | | 31 | 34 | 30 | 30·50 | 30·43 | | 10 | 10 | W | W | Clo. | | Fine |
| Jan. | | | | | | | | | | | | | | | |
| 1 | | | 31 | 36 | 32 | 30·48 | 30·51 | | 10 | 10 | W | WNW | Fog | | |
| 2 | | | 34 | 37 | 29 | 30·48 | 30·41 | | 10 | 11 | NE | SE | Fog | | |
| 3 | ○ | | 40 | 44 | 32 | 30·37 | 30·30 | | 10 | 9 | SW | SW | Fine | | Clo. |
| 4 | | | 35 | 39 | 35 | 30·19 | 30·12 | | 9 | 10 | SE | S | Clo. | Fine | Clo. |
| 5 | | | 37 | 41 | 34 | 30·17 | 30·22 | | 13 | 12 | SSE | SE | Fog | | Clo. |
| 6 | | | 36 | 39 | 39 | 30·11 | 30·01 | | 12 | 12 | S | S | Fog | Clo. | |
| 7 | | | 40 | 42 | 39 | 29·95 | 29·71 | | 11 | 12 | W | W | Fog | Rain | Clo. |
| 8 | | | 42 | 46 | 44 | 29·69 | 29·75 | | 15 | 16 | SW | SW | Fine | Rain | |
| 9 | | | 49 | 51 | 40 | 29·56 | 29·64 | | 15 | 17 | S | SW | Fine | Rain | Fine |
| 10 | | | 43 | 50 | 42 | 29·71 | 29·70 | | 13 | 12 | SW | S va. | Clo. | | Rain |
| 11 | ○ | | 45 | 49 | 41 | 29·76 | 29·87 | | 15 | 15 | SW | SW | Clo. | | Rain |
| 12 | | | 43 | 50 | 40 | 29·95 | 30·00 | | 16 | 15 | SW | W | Fine | Rain | Fine |
| 13 | | | 41 | 47 | 39 | 30·12 | 30·03 | | 17 | 15 | W | SSW | Fine | Rain | Fine |
| 14 | | | 44 | 50 | 40 | 29·93 | 29·99 | | 15 | 18 | SW | W | Rain | Clo. | |
| 15 | | | 45 | 49 | 44 | 30·10 | 30·00 | | 13 | 17 | S | SW | Clo. | Rain | |
| 16 | | | 46 | 48 | 39 | 30·22 | 30·05 | | 15 | 10 | SW | SW | Fine | | Clo. |
| 17 | | | 42 | 47 | 37 | 29·47 | 29·51 | | 15 | 10 | SW | SW | Rain | Fine | |
| 18 | | | 40 | 45 | 40 | 29·58 | 29·79 | | 11 | 13 | WSW | W | Fine | | |
| 19 | ○ | | 42 | 46 | 37 | 29·73 | 29·71 | | 10 | 10 | WSW | W | Fine | | |

**The probable quantity of rain (the gauge having froze and burst) fallen in December is 1 inch and 29-100ths.**

